# MicroRNA-26a inhibits wound healing through decreased keratinocytes migration by regulating ITGA5 through PI3K/AKT signaling pathway

**DOI:** 10.1042/BSR20201361

**Published:** 2020-09-29

**Authors:** Zhongping Jiang, Jie Wei, Weize Yang, Wen Li, Feng Liu, Xiaojie Yan, Xiaowei Yan, Niandan Hu, Jia Li

**Affiliations:** 1 Department of Emergency, Renmin Hospital of Wuhan University, Wuhan, China; 2 Department of Bone Surgery, Renmin Hospital of Wuhan University, Wuhan, China

**Keywords:** ITGA5, keratinocytes migration, miR-26a, wound healing

## Abstract

**Background:** Keratinocyte migration is essential for skin wound healing and recent studies demonstrated that microRNAs (miRNAs) are involved in the differentiation, migration and apoptosis in keratinocytes. However, the function of miR-26a in wound healing remains to be largely explored.

**Methods:** Northern blot and quantitative reverse transcriptase PCR (qRT-PCR) were used to detect the miR-26a expression and Western blot was used to detect integrin α-5 (ITGA5), phosphatidylinositol-3-kinase (PI3K), p-PI3K, protein kinase B (AKT) and p-AKT protein expression in immortalized human keratinocyte cell line HaCaT and normal human epidermal keratinocytes (NHEK) after 2 ng/ml transforming growth factor-β1 (TGF-β1) treatment for 0, 6, 12 and 24 h. Transwell assay and Wound healing assay were introduced to measure the cell migration of HaCaT cells. TargetScan online database, luciferase reporter assay and RNA immunoprecipitation (RIP) were employed to confirm the relationship between miR-26a and ITGA5.

**Results:** The RNA expression of miR-26a was down-regulated and ITGA5 protein expression was up-regulated by TGF-β1 treatment in HaCaT and NHEK cells in a time-dependent manner. MiR-26a overexpression inhibited the migration of HaCaT cells induced by TGF-β1 while miR-26a inhibitor enhanced the migration. ITGA5 was a downstream target mRNA and regulated by miR-26a. ITGA5 overexpression reversed the inhibitory effect of miR-26a on migration in HaCaT, while ITGA5 knockdown attenuated the stimulative effect of miR-26a inhibitor in HaCaT via PI3K/AKT signaling pathway.

**Conclusion:** MiR-26a overexpression inhibited TGF-β1 induced HaCaT cells migration via down-regulating ITGA5 through activating the PI3K/AKT signaling pathway.

## Introduction

Wound healing is a highly complex process which involves coagulation, inflammation, epithelialization, granulation tissue formation, matrix deposition and tissue remodeling [1]. Generally speaking, there are four sequential and overlapping phases during wound healing: hemostasis phase, inflammatory phase, proliferative phase and remodeling phase [2]. Among all of the phases, re-epithelialization which comprises proliferation and migration of keratinocytes plays a pivotal and essential role in wound healing [3]. In the process of re-epithelialization, epidermal keratinocytes from the wound margins proliferate and migrate to the wound and construct the new epithelium [4]. Particularly, re-epithelialization is largely regulated by transforming growth factor-β (TGF-β) which may modulate the migration of keratinocytes [5] but inhibits the cell proliferation [6]. However, the detailed mechanism of TGF-β remains to be further investigated.

MicroRNAs (miRNAs) are a group of non-coding RNAs which are approximately 18–23 nucleotides in length. Functionally, miRNAs bind to the 3′UTR of target mRNAs and inhibit the expression or mediate the degradation at post-transcriptional level [7]. MiRNAs play important roles in many biological processes, such as cell development, differentiation, proliferation and apoptosis [8]. Aberrantly expressed miRNAs are involved in many diseases, even carcinogenesis [9]. Recently, increasing studies have declared that miRNAs are participating in human skin repair and wound healing [10,11].

MiR-26a has been reported to play a tumor suppressor role in various cancers [12]. And recently, some studies suggested that miR-26a was related with wound healing. For example, Icli et al. claimed that miRNA-26a overexpression suppressed SMAD1 expression in endothelial cell and regulated pathological angiogenesis in mice [13]. In addition, inhibition of miR-26a could improve the angiogenesis and wound healing in diabetic db/db mice [14]. However, the underlying functional mechanism of miR-26a in wound healing is not clear.

Accumulating evidence has indicated that miR-26a regulated the various diseases progression via phosphatidylinositol-3-kinase (PI3K)/protein kinase B (AKT) signaling pathway. For example, miR-26a modulated PI3K/AKT signaling pathway in myocardial fibrosis [15]. In addition, miR-26a promoted angiogenesis after cerebral infarction through PI3K/AKT pathway [16]. Integrin α-5 (ITGA5) belongs to the integrin α chain family that plays critical roles in many cellular processes such as proliferation, viability and migration [17]. Several previous studies have presented that ITGA5 could operate in certain diseases including bladder cancer, oral squamous cell carcinoma and gastric cancer via PI3K/AKT pathway [18–20]. However, the interaction between miR-26a and ITGA5 in wound healing need to be further elucidated.

In the present study, we investigated the functional role of miR-26a in keratinocyte migration *in vitro*. Most important, we claimed that ITGA5 was an mRNA target of miR-26a. Our study implies that miR-26a overexpression inhibited keratinocyte migration via regulating ITGA5 expression during wound healing which provided a new insight into understanding the process of tissue repair.

## Methods

### Cell isolation and culture

Normal human epidermal keratinocytes (NHEK) were isolated as previously described [21]. In brief, NHEK were obtained from foreskins (18–30 years old, male, *n*=3) using Dispase II (Roche, U.S.A.) to remove the dermis from the epidermis followed by trypsinization and culture in EpiLife serum-free medium with 60 μM calcium supplemented with HKGS (Gibco, U.S.A.). All the patients read and signed the written informed consent and the experiments were approved by the Ethics Committee in Renmin Hospital of Wuhan University.

The immortalized human keratinocyte cell line HaCaT was obtained from China Center for Type Culture Collection (Wuhan, China), and HEK293T cells was purchased from ATCC. They were cultured in DMEM (HyClone, U.S.A.) supplemented with 10% fetal bovine serum. All the cells were maintained at 37°C incubator with 5% CO_2_ in a humid atmosphere.

For TGF-β1 treatment, NHEK or HaCaT cells were plated into six-well plate at the density of 5 × 10^4^/well and maintained for 24 h, after which they were serum-starved for another 24 h. Then, the cells were treated with 2 ng/ml TGF-β1 (R&D, U.S.A.) for 0, 6, 12 and 24 h.

### Northern blot

Total RNAs from cells were extracted from HaCaT and NHEK cells using TRIzol reagent (Sigma, T9424). The quality and concentration of RNA was determined by Nanodrop. The RNA samples were loaded carefully into the wells of agarose gel using pipettes. Then, the RNA was transferred on to a nylon membrane. The complementary strand of DNA (probe) was used to detect the RNA sequence that was present on the membrane. At last, the blot in a film cassette was placed and developed in the darkroom with X-ray film.

### Quantitative reverse transcriptase PCR

Total RNAs from cells were extracted using TRIzol reagent (Sigma, T9424). cDNA was reverse transcribed using miScript II RT Kit (Qiagen) for miR-26a and Reverse Transcription Reagents (Applied Biosystems) for U6. U6 small nuclear RNA (snRNA) was used to normalize miR-26a. QuantStudio™3 Real-Time PCR Systems (Thermo Fisher, U.S.A.) was used to detect the fluorescence of SYBR® Green (Promega, U.S.A.). The relative expression of miR-26a was calculated by the 2^−ΔΔ*C*_t_^ method. And the primers used were: miR-26a, forward 5′-GGATCCGCAGAAACTCCAGAGAGAAGGA-3′ and reverse 5′-AAGCTTGCCTTTAGCAGAAAGGAGGTT-3′; U6 forward 5′-GTTGACATCCGTAAAGACC-3′ and reverse 5′-GGAGCCAGGGCAGTAA-3′.

### Transient transfection

miR-26a mimic (sense sequence: 5′-TTCAAGTAATCCAGGATAGGCT-3′; antisense sequence: 5′-AGCCTATCCTGGATTACTTGAA-3′), miR-26a inhibitor (5′-AGCCTATCCTGGATTACTTGAA-3′), pcDNA-ITGA5, si-ITGA5 (5′-CTCCACAGATAACTTCACCCGAA-3′) and their corresponding negative controls were transfected into HaCaT cells using Lipofectamine 2000 (Invitrogen, U.S.A.) according to the instructions.

### Wound healing assay

Wound healing assay was utilized to examine the ability of cell migration. In brief, 1 × 10^5^ HaCaT cells were seeded into six-well plates. The next day, 10 μl pipette tips were performed to scratch the cell layer. Images were collected at 0 and 24 h, respectively, and the wound healing rate was calculated using ImageJ software.

### Luciferase assay

The sequence of ITGA5 3′UTR containing the putative miR-26a binding region was amplified from human genomic DNA. Then the sequence was cloned into pGL3 luciferase reporter vector (Promega, U.S.A.) (ITGA5 WT). The potential miR-26a binding sites were mutated by the Quick-Change Site-Directed Mutagenesis Kit (Agilent Technologies, U.S.A.) (ITGA5 MUT). The ITGA5 WT or ITGA5 MUT and miR-26a mimic were co-transfected into HEK293T cells by using Lipofectamine 2000 (Invitrogen, U.S.A.). The luciferase activity was measured using Dual-Luciferase Reporter Assay System (Promega, U.S.A.).

### RNA immunoprecipitation

RNA immunoprecipitation (RIP) assay was carried out using EZ-Magna RIP Kit (Millipore, U.S.A.) according to the protocols. Briefly, cells were lysed and incubated overnight at 4°C with protein A/G magnetic beads and anti-Ago2 (Millipore) or anti-IgG (Millipore) primary antibody. Then, excess DNA and protein in Ago2 or IgG immunoprecipitation were eliminated using RNase-free DNase I (Promega) and Proteinase K. Finally, miR-26a and ITGA5 mRNA enrichment degrees in Ago2 or IgG immunoprecipitation were determined by RT-qPCR assay.

### Western blot

Protein was extracted with RIPA lysis buffer following the protocol. Ten micrograms of protein of each sample was separated using SDS/PAGE and transferred to polyvinylidene fluoride (PVDF) membranes (Millipore, U.S.A.). After blocking with 5% dried non-fat milk in TBS, the membranes were incubated with specific primary antibodies ITGA5 (PA5-25433, 1:1000; Thermo Fisher, Waltham, MA, U.S.A.), PI3K (CY5355, 1:1000; Abways, Shanghai, China), AKT (#4685S, 1:1000; Cell Signaling Technology, Danvers, MA, U.S.A.), p-PI3K (CY6427, 1:1000; Abways), p-AKT (#4060S, 1:1000; Cell Signaling Technology) and β-actin (ab8227, 1:1000; Abcam, Cambridge, MA, U.S.A.) at 4°C overnight. Next day, the PVDF membrane was washed three times for 10 min each with Tris-buffered saline with Tween-20 (TBST) and incubated with HRP-conjugated IgG secondary antibody (1:2000 dilution, Santa Cruz Biotechnology Inc.) at room temperature for 1 h. After washing with TBST three times, the protein signals were detected using Pierce™ ECL Western blotting substrate (Thermo Fisher Scientific).

### Statistical analysis

All the data were analyzed using GraphPad Prism 7.0 (GraphPad Software, U.S.A.) and displayed as mean ± standard deviation (SD). For group comparison, Student’s *t* test was employed. *P*-value <0.05 was considered as statistically significant or as indicated.

## Results

### TGF-β1 down-regulated miR-26a in HaCaT and NHEK cells

**Figure 1:**
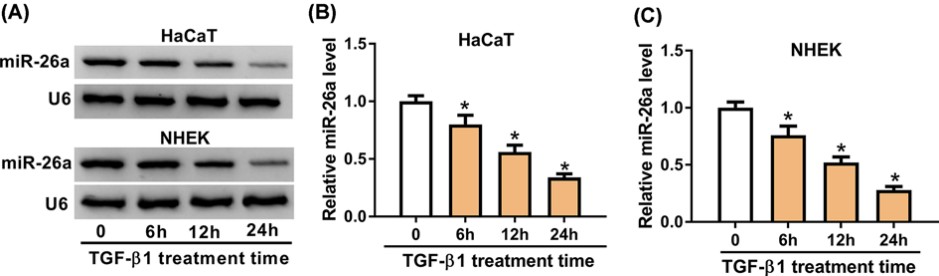
TGF-β1 inhibited miR-26a expression in HaCaT and NHEK cells (**A**) Representative Northern blot images of miR-26a in HaCaT and NHEK cells following 2 ng/ml TGF-β1 treatment for 0, 6, 12 and 24 h. (**B**) The miR-26a expression measured by qRT-PCR in HaCaT cells treated with 2 ng/ml TGF-β1 treatment for 0, 6, 12 and 24 h. (**C**) The miR-26a expression measured by qRT-PCR in NHEK treated with 2 ng/ml TGF-β1 treatment for 0, 6, 12 and 24 h. U6 was used as a control. * means *P*-value less than 0.05.

Previous studies have revealed that TGF-β signaling pathway plays an important role in the re-epithelialization by regulating keratinocyte migration and proliferation during wound healing [22,23]. So we used TGF-β1 to treat HaCaT and NHEK cells to mimic the process of wound healing *in vitro*. To verify the role of miR-26a in wound healing, we used 2 ng/ml TGF-β1 to treat HaCaT and NHEK cells for 0, 6, 12 and 24 h and checked its expression via Northern blot. As shown in Figure 1A, the mRNA level of miR-26a was gradually decreased from 6 h after treatment in a time-dependent manner. Next, we introduced quantitative reverse transcriptase PCR (qRT-PCR) to verify miR-26a expression. The miR-26a expression in 24 h was 0.34 ± 0.03-fold of that in 0 h in HaCaT cells after TGF-β1 treatment (Figure 1B). Meanwhile, we double-checked its expression in primary NHEK and found that miR-26a was significantly decreased (0.76 ± 0.08-fold) starting from 6 h and reached 0.28 ± 0.03-fold on 24 h after TGF-β1 treatment (Figure 1C). These results indicated that HaCaT cells treated with TGF-β1 was a satisfying model *in vitro* to mimic the primary epidermal keratinocytes under wound healing especially in the re-epithelialization and miR-26a was down-regulated in HaCaT and NHEK cells by TGF-β1 treatment in a time-dependent manner.

### Roles of miR-26a in TGF-β1-induced migration of HaCaT cells

**Figure 2:**
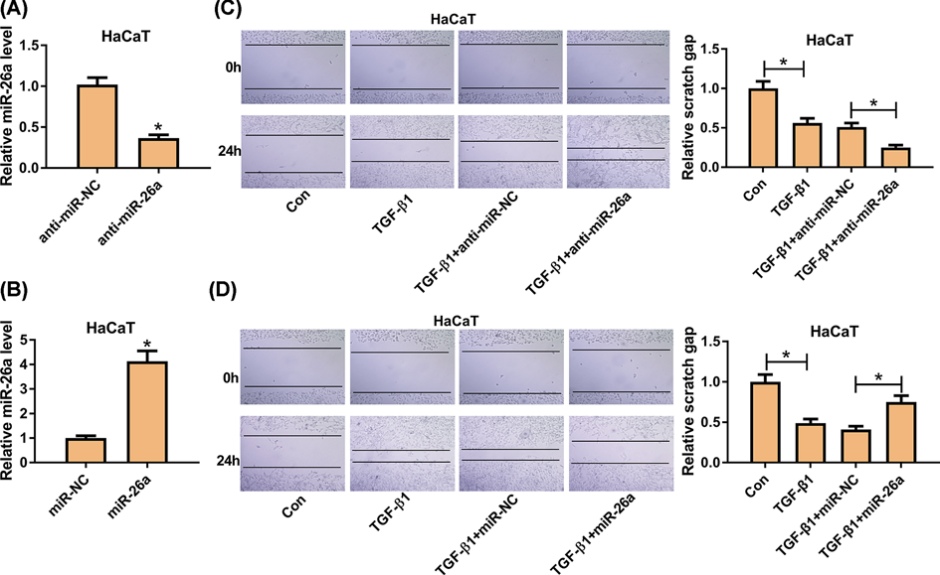
MiR-26a was involved in the migration of HaCaT cells (**A,B**) The level of miR-26a after transfection of anti-miR-26a or miR-26a was detected by qRT-PCR. (**C,D**) The migration ability of TGF-β1-treated HaCaT cells after transfection of anti-miR-26a was measured by wound healing assay. (B) The migration ability of TGF-β1-treated HaCaT cells after transfection of miR-26a was examined by wound healing assay. Con, no treatment. TGF-β1, treatment with 2 ng/ml TGF-β1 for 24 h. TGF-β1+anti-miR-NC, treatment with 2 ng/ml TGF-β1 and transfection with miR-26a inhibitor negative control. TGF-β1+anti-miR-26a, treatment with 2 ng/ml TGF-β1 and transfection with miR-26a inhibitor. TGF-β1+miR-NC, treatment with 2 ng/ml TGF-β1 and transfection with miR-26a negative control. TGF-β1+miR-26a, treatment with 2ng/ml TGF-β1 and transfection with miR-26a inhibitor. * means *P-*vvalue less than 0.05.

To explore the function of miR-26a in keratinocyte migration, we utilized gain- and loss-of-function analyses. First, we detected the transfection efficiency, and the results showed that miR-26a was significantly down-regulated in HaCaT cells by transfection of anti-miR-26a, and the expression of miR-26a was enhanced via transfecting with miR-26a (Figure 2A,B). As shown in Figure 2C, TGF-β1 elevated the number of migrated cells and reduced the scratch distance while miR-26a inhibitor compounded the migration in HaCaT cells. On the contrary, miR-26a overexpression attenuated the promote effect of TGF-β1 in cell migration (Figure 2D). These results indicated that miR-26a overexpression inhibited cell migration while silencing promoted the TGF-β1 induced migration of HaCaT cells.

### ITGA5 was a binding target of miR-26a

**Figure 3:**
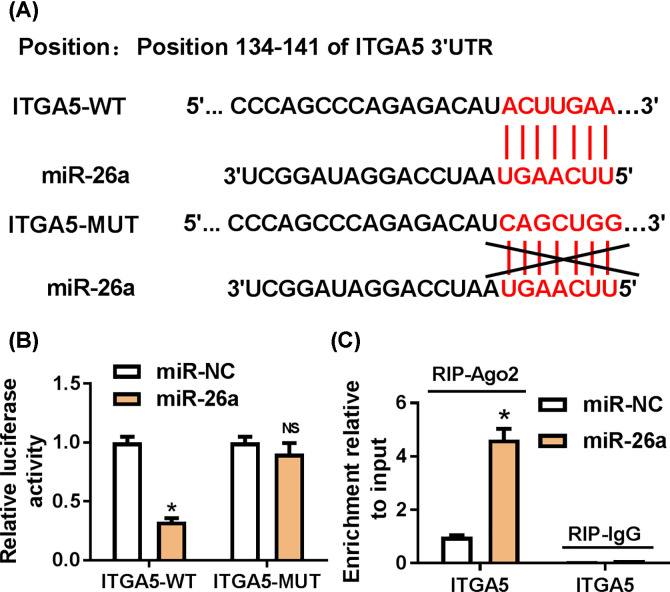
ITGA5 was a target of miR-26a (**A**) The potential binding sites between miR-26a and ITGA5 predicted with TargetScan tool. (**B**) The luciferase vector containing wildtype or mutant ITGA5 3′UTR was cotransfected into HEK293T with miR-26a. After 48 h, the luciferase activity was measured. (**C**) RIP assay of ITGA5 and miR-26 in HEK293T cells. * means *P*-value less than 0.05.

It has been proved that miRNAs may play function in gene expression via inhibiting translation or mediating degradation through binding to the 3′UTR of specific mRNA [24]. Thus, we used TargetScan tool to predict the mRNA target of miR-26a. As shown in Figure 3A, the position of 134–141 in 3′UTR of ITGA5 was complementary to miR-26a. Then, we constructed luciferase reporter vector containing wildtype 3′UTR ITGA5 or mutant 3′UTR ITGA5 (Figure 3A) and transfected the vector into HEK293T cells with miR-26a. The luciferase activity was significantly decreased when co-transfecting wildtype 3′UTR ITGA5 and miR-26a, but no change with mutant 3′UTR ITGA5 (Figure 3B). Next, we performed RNA immunuprecipitation assay to confirm the relationship between ITGA5 and miR-26a and ITGA5 mRNA was enriched in the Ago2 precipitation (Figure 3C). Together, these results suggested that ITGA5 was a binding target of miR-26a.

### ITGA5 was up-regulated by TGF-β1 via miR-26a

**Figure 4:**
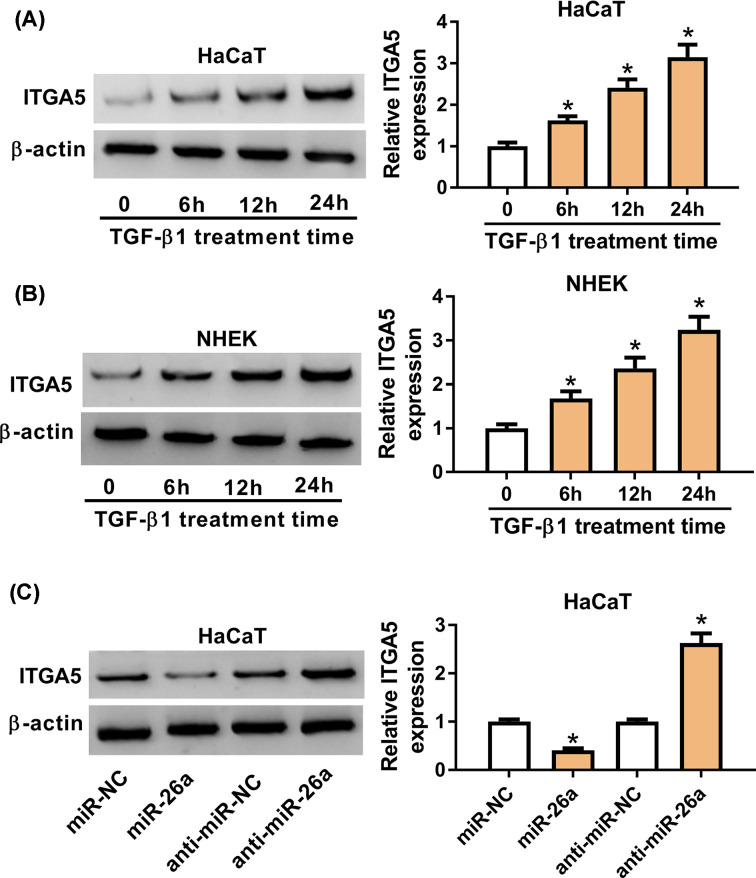
ITGA5 was up-regulated by TGF-β1 and modulated by miR-26a (**A**) HaCaT cells were treated with 2 ng/ml TGF-β1 for 0, 6, 12 and 24 h. The ITGA5 expression in the treated HaCaT cells was detected by Western blot assay. (**B**) ITGA5 protein expression of NHEK after 2 ng/ml TGF-β1 treatment for 0, 6, 12 and 24 h was detected by Western blot assay. (**C**) ITGA5 protein expression in HaCaT cells after transfection of miR-26a inhibitor or miR-26a was measured by Western blot assay. β-actin was used as reference for ITGA5. * means *p*-value less than 0.05.

Considering that ITGA5 was a target mRNA of miR-26a, we further explored the role of ITGA5 in TGF-β1 treatment. So we continually used 2 ng/ml TGF-β1 to treat HaCaT cells. The protein expression of ITGA5 was gradually increased from 6 h after TGF-β1 stimulation and was approximately three-folds on 24 h (Figure 4A). Meanwhile, ITGA5 expression was up-regulated in NHEK (Figure 4B). To further understand the relationship between ITGA5 and miR-26a, we overexpressed miR-26a in HaCaT cells and ITGA5 was down-regulated, while miR-26a inhibitor could promote the expression of ITGA5 (Figure 4C). These results indicated that TGF-β1 could regulate ITGA5 expression via miR-26a.

### MiR-26a was involved in migration of TGF-β1-treated HaCaT cells via ITGA5 through regulating PI3K/AKT signaling pathway

**Figure 5:**
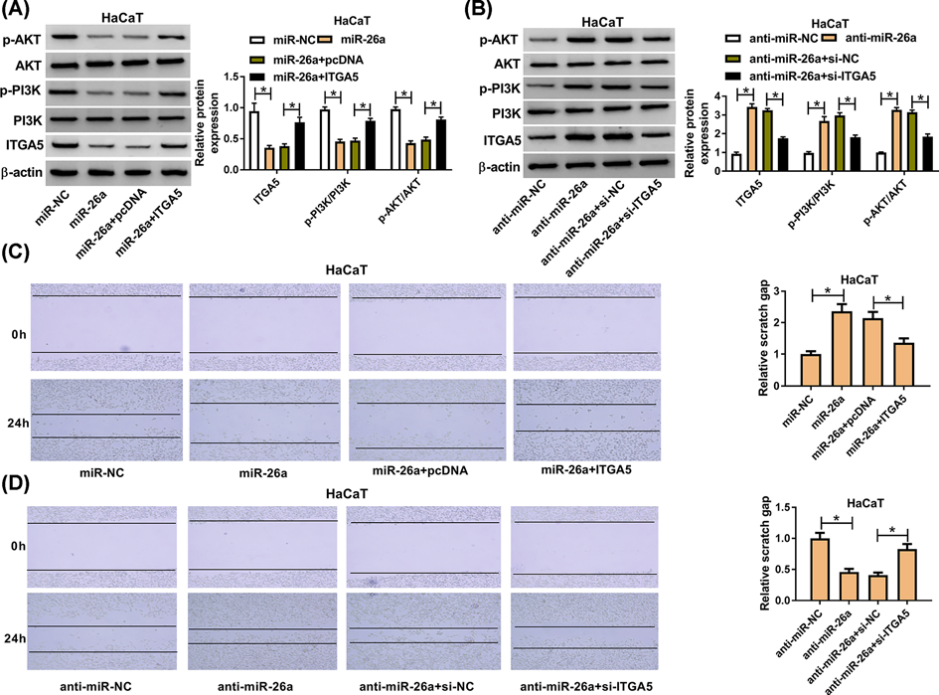
ITGA5 reversed the effect of miR-26a on cell migration via PI3K/AKT signaling pathway (**A**) The expression level of ITGA5 was detected by Western blot assay after transfection with miR-26a or miR-26a+ITGA5 in HaCaT cells. (**B**) The expression level of ITGA5, PI3K and AKT was measured after transfection with anti-miR-26a or anti-miR-26a+si-ITGA5. (**C,D**) The migration ability of HaCaT cell was analyzed after overexpressing both miR-26a and ITGA5 in HaCaT cells after TGF-β1 treatment for 24 h. (B) The migration cell number of HaCaT after transfection of miR-26a inhibitor and si-ITGA5 after TGF-β1 treatment for 24 h. * means *P*-value less than 0.05.

Given that ITGA5 was a target of miR-26a and miR-26a was involved in the migration of TGF-β1-treated HaCaT cells, we further investigated the role of ITGA5 in keratinocyte migration. We co-transfected miR-26a and pcDNA-ITGA5 into HaCaT cells which had the treatment of TGF-β1 for 24 h and found that miR-26a overexpression down-regulated the expression of ITGA5, p-PI3K and p-AKT, while the ITGA5 restored the inhibitory effect of miR-26a on ITGA5, p-PI3K and p-AKT expression. On the contrary, ITGA5 silencing via small interfering RNA had the function of reversing the promotion effect of miR-26a inhibitor on ITGA5, p-PI3K and p-AKT expression in TGF-β1-stimulated HaCaT cells (Figure 5A,B). In addition, ITGA5 overexpression reversed the inhibitory role of miR-26a in migration of HaCaT after TGF-β1 treatment for 24 h (Figure 5C). Previous data demonstrated that miR-26a inhibitor could promote the migration, but knockdown of ITGA5 attenuated the migration caused by miR-26a inhibitor (Figure 5D). These data indicated that miR-26a could regulate the keratinocyte migration via modulating ITGA5 through PI3K/AKT signaling pathway.

## Discussion

Wound healing is a complex process in multicellular organisms which comprises lots of signaling pathways in skin compartments and the extracellular matrix (ECM). The process of wound healing can be divided into four sequential phases: hemostasis, inflammation, proliferation and remodeling [25]. Disruption of any phase may lead to development of chronic wound or formation of a keloid. It is rather remarkable that growth factor, TGF-β is involved in all the phases of the repair process [26,27].

TGF-β superfamily contains four highly conserved isoforms (TGF-β1–4). All of the isoforms have an N-terminal signal peptide of 20–30 amino acids which is required for secretion, a pro-region called latency-associated peptide (LAP) and a C-terminal region that becomes the mature TGF-β molecule. Under proteolytic cleavage, the mature TGF-β is released from LAP [28], and then binds to its receptor, activating the TGF-β signaling pathway [29]. It has been suggested that this pathway is involved in development, tissue regeneration and immune responses. The aberrant expressions of TGF-β always happen in cancers and fibro-proliferative disorders [30]. As mentioned above, TGF-βs are involved in all phases of skin wound healing. After injury, the TGF-β could be released by platelets and recruits innate immune cells and attracts fibroblasts to stimulate the proliferation [5]. However, the detailed mechanism of TGF-β in wound healing is still largely unknown. In order to understand the role of TGF-β, we used TGF-β1 to treat the keratinocyte to mimic the wound healing *in vitro*. We adapted two kinds of keratinocytes, one is NHEK which were obtained from foreskins, the other one is immortalized human keratinocyte cell line, HaCaT. Under the treatment, the cells migration ability was promoted which is an important feature in wound healing. After treatment with 2 ng/ml TGF-β1, we first found that miR-26a was gradually decreased via Northern blot and qRT-PCR. However, it is still unknown how TGF-β1 regulated the expression of miR-26a, thus the further study is to explore the regulatory mechanism of TGF-β1 on miR-26a.

MiR-26a belongs to the miR-26 family (miR-26a, miR-26b) which regulates a large number of signaling pathways including PI3K/AKT signaling pathway and various cellular processes such as cell proliferation, migration and differentiation. Dysregulated miR-26 family plays a central role in many diseases. For example, miR-26a is essential for skeletal muscle differentiation and regeneration via regulating the expression and activity of Smad1 and Smad4 [31]. In cardiac hypertrophy, miR-26a was down-regulated while overexpression of miR-26a can inhibit the process of myocardial hypertrophy via decreasing GSK3β expression [32]. Besides, increasing studies have suggested that miR-26a is dysregulated in many cancers and is related with differentiation, proliferation, invasion, angiogenesis and chemoresistance [33]. In most cancers, miR-26a serves as a tumor suppressor to inhibit the expression of target oncogenes. However, there are few studies on the function of miR-26a in wound healing. Icli et al. demonstrated that miR-26a increased approximately 3.5-times in the punch skin biopsy wound of db/db mice. Anti-miR-26a could induce angiogenesis, increased granulation tissue thickness and accelerated wound closure via regulating the target gene, *SMAD1* [14]. In our present work, we found that miR-26a overexpression inhibited HaCaT cell migration after TGF-β1 treatment while anti-miR-26 could promote the cell migration of TGF-β1-induced HaCaT cells. Next, we predicted the ITGA5 was an mRNA target of miR-26a via bioinformatics analysis. Then we confirmed the relationship between miR-26a and ITGA5 using dual-luciferase reporter assay and RIP. Moreover, we proposed that miR-26a could negatively regulate the expression of ITGA5.

ITGA5, also called ITGA5, was a member of the integrin α chain family. Integrins are transmembrane receptors that facilitate cell–ECM adhesion. Once ligand binds to the integrin, it can activate signal transduction pathways which can regulate cell cycle, cytoskeleton organization and etc [34]. Basically speaking, there are two main functions of integrins: attachment of the cell to the ECM and signal transduction from the ECM to the cell. The integrins are also involved in many biological process such as immune patrolling, cell migration, adhesion [35]. Emerging evidences have revealed that integrin is related with keratinocyte migration. Integrin β1 knockout mouse displayed reduced proliferation, loss of sebaceous glands and hair follicles, and impaired wound re-epithelialization [36,37]. Like other integrins, some are persistent or have enhanced expression such as α3β1, α6β4, α2β1, α9β1 and αvβ5; some are expressed *de novo* such as α5β1 and αvβ6 [38]. These results indicated that the integrin members may display different functions in the wound healing. In our study, we found that ITGA5 was up-regulated after TGF-β1 treatment. And overexpression of ITGA5 could increase the migration ability which was inhibited by miR-26a mimic, while ITGA5 silencing attenuated the migration induced by miR-26a inhibitor. In addition, previous findings showed that ITGA5 regulated PI3K/AKT signaling pathway in oral squamous cell carcinoma [39]. Consistent with previous study, we found that ITGA5 regulated PI3K/AKT signaling pathway in TGF-β1-treated HaCaT cells. And interestingly, ITGA5 reversed the changes of PI3K/AKT pathway-related proteins expressions by miR-26a in HaCaT cells. Thus, miR-26a inhibits keratinocytes migration by targeting ITGA5 through PI3K/AKT signaling pathway in wound healing. In conclusion, we verified that TGF-β1 could promote the migration of keratinocyte cell which was consistent with previous study. And TGF-β1 could down-regulate the expression of miR-26a as well as increase ITGA5 expression. However, anti-miR-26a promoted cell migration while miR-26a mimic inhibited the migration. In addition, ITGA5 was a target of miR-26a and regulated by miR-26a. Notably, ITGA5 overexpression could reverse the inhibitory effect of miR-26a in migration and ITGA5 silencing inhibited the migration induced by miR-26a inhibitor. Taken together, miR-26a could inhibits wound healing through decreased keratinocytes migration by regulating ITGA5 which provides a new insight into understanding the miR-26a in tissue repair and miR-26a/ITGA5 could be a potential therapeutical target to heal the wound.

## Abbreviations

AKT: protein kinase B
ECM: extracellular matrix
ITGA5: integrin α-5
LAP: latency-associated peptide
miRNA/miR: microRNA
NHEK: normal human epidermal keratinocyte
PI3K: phosphatidylinositol-3-kinase
PVDF: polyvinylidene fluoride
qRT-PCR: quantitative reverse transcriptase PCR
RIP: RNA immunoprecipitation
TBST: Tris-buffered saline with Tween-20
TGF-β: transforming growth factor-β
